# Clinical features and treatment of apoplectic intratumoral hemorrhage of glioma

**DOI:** 10.1186/s12883-024-03753-6

**Published:** 2024-07-24

**Authors:** Jia-hua Zhou, Chao Wang, Di Yang, Ying-xi Wu, Da-yun Feng, Huaizhou Qin, Ju-lei Wang, Ming-hao Wei

**Affiliations:** 1 Department of Neurosurgery, Tangdu Hospital, Air Force Medical University, No. 569 Xinsi Road, Baqiao District, Xi’an, Shanxi 710038 China; 2 Department of Radiology, Tangdu Hospital, Air Force Medical University, Xi’an, Shanxi 710038 China

**Keywords:** Apoplectic intratumoral hemorrhage (AIH), Apoplectic intratumoral hemorrhage of glioma (AIHG), Apoplexy, Chemotherapy, GBM, Glioblastoma, Glioma hemorrhage, Glioma stoke, Radiotherapy, Simple cerebral hemorrhage

## Abstract

**Objective:**

The primary objective of this study was to explore the clinical characteristics of apoplectic intratumoral hemorrhage in gliomas and offer insights for improving the diagnosis and treatment of this disease.

**Methods:**

We analyzed the clinical data of 35 patients with glioma and hemorrhage. There were eight cases of multiple cerebral lobe involvement, and 22 cases involved a single lobe. Twenty-one patients had a preoperative Glasgow Coma Scale (GCS) score of ≥ 9 and had a craniotomy with tumor resection and hematoma evacuation after undergoing preoperative preparation. A total of 14 patients with GCS < 9, including one with thalamic hemorrhage breaking into the ventricles and acute obstructive hydrocephalus, underwent craniotomy for tumor resection after external ventricular drainage (EVD). One patient had combined thrombocytopenia, which was surgically treated after platelet levels were normalized through transfusion. The remaining 12 patients received immediate intervention in the form of craniotomy hematoma evacuation and tumor resection.

**Results:**

We performed subtotal resection on three tumors of thalamic origin and two tumors of corpus callosum origin, but we were able to successfully resect all the tumors in other locations that were gross total resection Pathology results showed that 71.43% of cases accounted for WHO-grade 4 tumors. Among the 21 patients with a GCS score of ≥ 9, two died perioperatively. Fourteen patients had a GCS score < 9, of which eight patients died perioperatively.

**Conclusions:**

Patients with a preoperative GCS score ≥ 9 who underwent subemergency surgery and received aggressive treatment showed a reasonable prognosis. We found their long-term outcomes to be correlated with the pathology findings. On the other hand, patients with a preoperative GCS score < 9 required emergency treatment and had a high perioperative mortality rate.

## Introduction

Glioma is the most common intracranial malignant tumor. Among these, some tumors, especially high-grade gliomas, often present with coexisting apoplectic intratumoral hemorrhage. They have an acute onset, are severe in most cases, and are easily misdiagnosed, posing significant challenges in the diagnosis and treatment of the disease. There are only a few current studies of the disease, mostly described as case reports or addressed radiographically [1–3].

In this paper, we examined data from 35 cases of patients who underwent surgery at the Air Force Medical University and were diagnosed in the pathology investigations as having definite gliomas complicated by apoplectic intratumoral hemorrhage (AIH) between August 2010 and August 2023. We analyzed the clinical features based on a review of the relevant literature and summarized the treatment experience. This study was approved by the ethics committee of the hospital, and consent was obtained from the patient’s family. The study received the approval of the institutional review board of the Tel Aviv Medical Center. We have presented the literature review and our insights on the management of these lesions in the following sections:

## Materials and methods

### Clinical data

We examined the clinical data of 35 patients with apoplectic intratumoral hemorrhage of glioma (AIHG) diagnosed by pathology who were admitted to the Tangdu Hospital affilitaed Air Force Medical University between August 2010 and August 2023. There were 18 males and 17 females. The age range was 11–73 years, with a mean of 59.14 years. Six patients in this group had a history of previous glioma resections, and the remaining 29 patients were diagnosed for the first time.In this group, 3 patients were complicated with hypertension, and 1 patient underwent mastectomy 8 years ago. The remaining patients had no history of systemic diseases. Among the patients in this group, 1 patient had thrombocytopenia and underwent surgery after platelet transfusion to the normal range. None of the patients took aspirin, warfarin and other anticoagulants.

### Clinical features

The chief complaints at the time of admission for this group of patients were as follows: 18 patients reported a sudden-onset headache with nausea and vomiting; 11 patients had sudden exacerbations with a previous history of headache; four patients reported sudden headaches with limb or speech dysfunction on one side; and two patients had sudden seizures.

*We used the* Glasgow Coma Scale (GCS) *after intratumoral hemorrhage of glioma to describe the consciousness level of this group of patients* [4]. Within 24 h of onset, 21 patients were lucid and had a Glasgow Coma Scale (GCS) score of ≥ 9; one patient suffered a tumor hemorrhage again during preparation, and she was unconscious with a GCS score of < 9 points, requiring emergency surgery.

Among the 14 patients who were unconscious within 24 h of admission and had a GCS score less than 9, one patient with a cystic glioma and hemorrhage underwent external drainage through cystic fluid puncture. This patient regained lucidity and recovered to a state of wakefulness, achieving a GCS score of 15. Subsequently, elective surgery was performed.

There were 21 patients with a preoperative GCS score of ≥ 9 points and 14 patients with a GCS score of < 9 points. We used the Karnofsky Performance Scale (KPS) to evaluate health status after glioma resection [5]. The calculation of GCS scores and Karnofsky Performance Scale (KPS) scores was conducted using information available in the medical case files. KPS scores were assigned either based on the provider’s report or retrospectively by one of the researchers, relying on information extracted from the medical records.

### Preoperative imaging

All patients in this study underwent cranial CT preoperatively, which revealed clumps or scattered hyperdense hemorrhagic foci with heavy edema surrounding the hemorrhagic foci. All lesions were found to be supratentorial, with infiltration in multiple lobes in 8 cases, temporo-parietal in 4 cases, parieto-occipital in 2 cases, fronto-temporal insula in 1 case, and fronto-parietal in 1 case. There were 22 patients with a single-lobe involvement, including 11 with frontal, 7 with temporal, 3 with parietal, and 1 with occipital lobes. Three cases had a tumor of thalamic origin, and in two cases, the origin was in the corpus callosum. Tumor hemorrhages broke into the ventricular system in 5 cases and formed acute hydrocephalus in 1 case. Among them, one case of cystic glioma with hemorrhage showed hemorrhage in the cavity of the tumor capsule; the lower part was high-density blood, and the upper part was low-density cystic fluid. The rest were solid gliomas with apoplectic intratumoral hemorrhage (AIH).

Among the patients in our cohort, 19 underwent cranial MR plain + contrast-enhanced scanning. Irregular shape of hematoma, uneven density, and relatively diffuse hemorrhage were seen, enhancing obvious tumor lesions were seen on enhanced cranial MR, and peritumoral edema was obvious. In 1 patient with intraventricular hemorrhage, a whole brain angiogram was performed, and the vessels showed no abnormalities.

### Diagnosis and treatment

There were 5 patients who were initially diagnosed in primary hospitals with “intracerebral hemorrhage”, of whom 1 received conservative treatment, 2 underwent external drainage of hematoma by hematoma puncture, and 2 others underwent craniotomy hematoma evacuation + decompressive debridement of the bone flap. Of these 5 patients, 3 had second hemorrhages, 1 patient had postoperative headache without remission and underwent a cranial MRI that indicated glioma, and 1 patient with a skull defect needed to have cranial defect repair. All of them were admitted to our department for further treatment and subsequently diagnosed as having AIH. The other 30 patients in our study were first diagnosed with AIH in our hospital.

Twenty-one patients with a preoperative GCS of ≥ 9 points were admitted and received symptomatic treatment including dehydration, prevention of epilepsy, and hemostasis, among other interventions. They underwent a comprehensive examination that included plain + enhanced cranial MRI, and surgery was performed as soon as possible. Surgical flaps and approaches were routinely planned as per the tumor site. The extent of the tumor was determined as per the imaging data and localized under intraoperative ultrasound guidance and microscopic view. We attempted a total resection of the tumor based on the objective of protecting the integrity of the normal surrounding functional areas. In our procedures, intraoperative ultrasonic exploration was employed to confirm the absence of tumor residue while simultaneously addressing the removal of hematoma and contused brain tissue. Close attention was given to hemostasis, ensuring the successful completion of the surgery. Notably, in this cohort, all bone flaps were routinely repositioned during the initial craniotomy, except in cases where such flaps had been previously excised prior to surgery.

In the study cohort, there were 14 patients who had a preoperative GCS of < 9 points. One patient who had an acute obstructive hydrocephalus due to a thalamic hemorrhage breaking into the ventricle underwent ventricular puncture and external drainage, and then a craniotomy to remove the tumor and hematoma. One patient with thrombocytopenia was treated with a platelet transfusion to bring the counts back to normal and then underwent surgery. The other 12 patients underwent emergency craniotomy procedures for hematoma evacuation and tumor resection.

The decision to perform decompressive craniectomy was determined by assessing both preoperative pupillary response and intraoperative intracranial pressure. If, prior to surgery, there was dilation of one pupil and high intracranial pressure, a decompressive craniectomy was performed. Three patients underwent glioma resection + hematoma evacuation + decompressive craniectomy. Tracheotomy was performed for patients in coma and those with difficulty in expectoration after the surgery. A total of eight patients underwent tracheotomy procedures.

### Statistical analysis

We used SPSS 26.0 software for statistical analysis. Comparisons between groups were done using the Chi-square test.

## Results

### Surgical findings

In all the initial surgeries, we observed higher intraoperative intracranial pressure, with the exception of the following four cases: one patient who had undergone external drainage via ventricular puncture before surgery, one who had undergone external drainage via cyst cavity puncture, and two who had undergone craniotomy with hematoma evacuation and decompressive craniectomy. Evident within these observations is the presence of dark red blood clots or liquefied dark red blood within the tumor. Microscopically, a solid part of the tumor with a distinct texture from the surrounding normal brain tissue was observed. We performed a subtotal resection on three tumors of thalamic origin and two of corpus callosum origin, and the remaining parts of the tumor were completely excised with no residual mass left behind.

### Pathology results

Pathology results for this group of patients revealed the following types of tumors: 1 oligodendroglioma of WHO-grade 2; 4 oligodendrogliomas of WHO-grade 3; 5 astrocytomas of WHO-grade 3; 23 glioblastomas; and 2 gliosarcomas. WHO-grade 4 tumors accounted for 71.43%. Eight patients had recurrent glioma, and among them, 1 case was diagnosed as oligodendroglioma grade 2 in the first surgical pathology evaluation, and the pathological result after recurrence was anaplastic oligodendroglioma grade 3. Two cases were astrocytoma grade 2, and the pathology results were glioblastoma after recurrence. One case of oligodendroglioma was of WHO-grade 3, and the pathology diagnosis was glioblastoma after recurrence. One case of astrocytoma was of WHO-grade 3, and the pathology results were unchanged after recurrence. Two cases of glioblastomas and one case of gliosarcoma had no pathological changes after recurrence.

### Complications and prognosis

We classified deaths resulting from any complications caused by surgery-related factors within one month of surgery as surgery-related deaths [6]. In this cohort, there were 21 patients with a preoperative GCS score of ≥ 9, and 2 died perioperatively. Another patient in postoperative coma due to thalamic glioma, died of a pulmonary infection. The other death was of a patient with frontal lobe glioma who developed rebleeding after surgery and required a secondary surgical craniotomy to remove the hematoma and decompressive debridement of the bone flap, but it was not lifesaving.

The remaining 19 cases were successfully treated and discharged with an average KPS score of 80.53 (ranging from 40 to 90) (Table 1). Among the 14 patients with a preoperative GCS score of < 9, 8 died perioperatively due to prolonged coma in all cases. Among them, 7 patients developed pulmonary infections, and 1 patient had an intracranial infection, ultimately leading to systemic multiple organ failure. The remaining 6 patients managed to survive the perioperative period, with an average KPS score of 61.67 (ranging from 40 to 90) (Table 2). The perioperative mortality in the group with a preoperative GCS score of ≥ 9 was significantly lower than that in the group with a GCS score of < 9 (*P* = 0.02).

**Table 1:** Clinical data of 21 patients with preoperative GCS ≥ 9

Case	Sex	Age	Glioma history/Medical history	Initial diagnosis and therapies	Location of glioma	EOR of primary glioma	Complications	Histological doagnosis	KPS (1month after operation)	Subsequent therapy	OS from initial diagnosis (mon)
1	M	58	6y, LGA/NO	AIHG	Right frontal lobe	GTR	Surgical area hemorrhage	GBM	0	NULL	NULL
2	M	48	NO/NO	CH; HE + DC	Left parietal lobe	GTR + CRA	NO	AA	50	NULL	9
3	F	30	NO/NO	CH; CT	right temporal lobe	GTR	NO	AA	90	RT + TMZ	18
4	F	27	NO/NO	AIHG	right temporal lobe	GTR	NO	AA	90	RT + TMZ	26
5	M	55	NO/NO	CH; CT	callosum	PR	PI + intracranial infection	GBM	40	NULL	3
6	M	20	NO/NO	CH; HE + DC	Left parietal lobe	GTR	hemiplegia of the right limb	GBM	70	NULL	2
7	F	52	NO/NO	AIHG	Right temporal lobe	GTR	NO	GBM	90	NULL	6
8	F	56	NO/NO	AIHG	Left occipital lobe	GTR	NO	AA	90	RT + TMZ	14
9	M	57	NO/HTN	AIHG	Right temporal lobe	GTR	NO	GBM	90	RT + TMZ	9
10	F	56	NO/NO	CH broke into the ventricle;	Left thalamus	PR	NO	GBM	50	NULL	3
11	F	14	8Mon, GBM, PR/NO	AIHG	Left thalamus	PR + TRA	PI + thrombus of lower extremity veins	GBM	0	NULL	NULL
12	F	60	NO/HTN	AIHG	Right parieto - occipital lobe	GTR	NO	GBM	90	RT + TMZ	15
13	F	39	NO/NO	AIHG	Left frontal lobe	GTR	NO	gliosarcoma	90	RT + TMZ	5
14	F	36	NO/NO	Cystic glioma with hemorrhage, cystic fluid puncture and external drainage	Right frontal lobe	GTR	NO	AO	90	RT + TMZ	36*RF
15	F	61	8Mon, GBM, GTR/HTN	AIHG	Left frontal lobe	GTR	NO	GBM	80	RT + TMZ	26
16	M	54	NO/NO	AIHG	Left frontal lobe	GTR	NO	AO	90	RT + TMZ	8* RF
17	M	56	NO/NO	AIHG	Left frontotemporal parietal lobe	GTR	NO	GBM	90	RT + TMZ	5* RF
18	F	16	NO/NO	AIHG	Left frontoparietal lobe	GTR	NO	AA	80	RT + TMZ	15(12 months RE, reoperation,3 monsdie )
19	F	11	NO/NO	AIHG	Right frontal lobe	GTR	NO	GBM	90	RT + TMZ	4* RF
20	M	58	NO/NO	AIHG	Left parietal lobe	GTR	NO	GBM	80	RT + TMZ	11
21	M	50	NO/NO	CH; HE	Left temporal parietal lobe	GTR	NO	GBM	90	RT + TMZ + BEA	12* RE

M, male; F, female; GBM, glioblastoma; Mon, month; Y, year; LGA, low grade astroma; AIHG, apoplectic intratumoral hemorrhage of glioma; CH, cerebral hemorrhage; CRA, Craniotomy; CT, conservative treatment; DC, decompressive craniectomy; HE, hematoma evacuation; AA, Anaplastic astrocytoma; BEA, bevacizumab; OS, overall survival; AO, anaplastic oligodendroglioma; RE, recurrence; RF, relapse-free; PI, pulmonary infection; CRA, Craniotomy; TRA, tracheotomy.HTN, hypertension

*The patient remained alive at the end of follow-up

**Table 2:** Clinical data of 14 patients with preoperative GCS < 9

Case	Sex	Age	Glioma history/Medical history	Initial diagnosis and therapies	Location of glioma	EOR of primary glioma	Complications	histological doagnosis	KPS (1month after operation)	Subsequent therapy	OS from initial diagnosis (mon)
1	M	38	NO/NO	Hemorrhage breaks into ventricular; hydrocephalus; Intraventricular puncture and external drainage	Left thalamus	PR + TRA	Fever + coma + PI	GBM	0	NULL	NULL
2	M	46	NO/NO	AIHG	Left frontal lobe	GTR	NO	GBM	70	NULL	5(2 months RE, GTR,3 months die )
3	F	52	NO/NO	AIHG	Right parieto-occipital lobe	GTR + CRA	NO	GBM	60	NULL	8
4	M	42	7Y, AA/NO	AIHG	Right frontal lobe	GTR + TRA	coma + PI	GBM	0	NULL	NULL
5	M	25	NO/NO	AIHG	callosum	PR + Lumbar drainage	Hydrocephalus + intracranial infection + coma	GBM	0	NULL	NULL
6	M	39	NO/NO	AIHG	Right temporal parietal lobe	GTR	NO	GBM	50	NULL	4
7	F	57	NO/NO	AIHG	Right temporal lobe	GTR + TRA	Decompressive craniectomy was performed for cerebral edema	GBM	40	NULL	3
8	M	62	1 Y, GBM/NO	AIHG	Left frontotemporal insula	GTR	persistent coma; PI	GBM	0	NULL	NULL
9	M	53	NO/NO	AIHG	Right temporal lobe	GTR	coma; PI	GBM	0	NULL	NULL
10	M	54	5Y, LGO/NO	AIHG	Right frontal lobe	GTR + CRA	Coma, PI	AO	0	NULL	NULL
11	F	72	NO/Breast cancer postoperative 8 years	AIHG	Right temporal parietal lobe	GTR + TRA	NO	gliosarcoma	90	NULL	2months RE, GTR, Coma, Pulmonary infection, Death occurred during perioperative period
12	F	73	NO/NO	AIHG, Low platelet s, platelet transtasion	Left frontal lobe	GTR + TRA	Coma, PI	GBM	0	NULL	NULL
13	F	32	NO/NO	AIHG	Left frontal lobe	GTR + TRA	NO	LGO	60	RT + TMZ	29*
14	M	71	NO/NO	AIHG	Left temporal insula	GTR + CRA + TRA	Coma, PI	GBM	0	NULL	NULL

M, male; F, female; GBM, glioblastoma; Mon, month; Y,year; LGA, low grade astroma; AIHG, apoplectic intratumoral hemorrhage of glioma; CH, cerebral hemorrhage; CRA, Craniotomy; TRA, tracheotomy; CT, conservative treatment; DC, decompressive craniectomy; HE, hematoma evacuation; AA, Anaplastic astrocytoma; BEA, bevacizumab; LGO, Low grade oligodendrocy; AO, anaplastic oligodendroglioma; GTR, gross total resection; PR, partial resection; SRS, stereotactic radiosurgery; TMZ, temozolomide; RT, radiotherapy; Re-op, reoperation; Bev, bevacizumab; OS, overall survival; RE, recurrence; RF, relapse-free; PI, pulmonary infection

*The patient remained alive at the end of follow-up; RE, recurrence; RF, relapse-free; PI, pulmonary infection

We classified gliomas originating from the thalamus or corpus callosum as deep-seated gliomas. In our series, 3 patients with thalamic origin and 2 patients with corpus callosum origin underwent major partial resection. Among them, 3 died perioperatively, while the other 2 patients died three months after surgery.

### Postoperative treatment and follow-up

Among the patients with a preoperative GCS score of ≥ 9 points, 19 patients were cured, and the pathology results were as follows: there were 11 cases of glioblastoma, 1 case of gliosarcoma, 5 cases of anaplastic astrocytoma of WHO-grade 3, and 2 cases of anaplastic oligodendroglioma of WHO-grade 3. Among them, 14 received postoperative concurrent chemoradiotherapy (temozolomide and bevacizumab), and five did not undergo subsequent treatment. As of date, there were 14 deaths (one of which recurred 12 months after surgery and the patient died three months after the second surgery), with a mean survival time of 11.57 months (2–26 months); five patients are alive, among whom one relapsed and four are recurrence-free, with a mean follow-up time of 13 months (4–36 months) (Table 1).

Of the 14 patients with a preoperative GCS score of < 9, 6 were cured, and the pathology results were 4 cases of glioblastoma, 1 case of gliosarcoma, and 1 case of oligodendroglioma of WHO-grade 2. As of date, 5 of them with high-grade glioma died without postoperative radiotherapy or chemotherapy (2 of them underwent a second surgical resection after tumor recurrence; 1 patient died during the perioperative period due to recurrence 3 months later; 1 patient had a recurrence 2 months later and underwent a reoperation but died 5 months after the second operation). The mean survival time was 4 months (2–8 months). One patient with oligodendroglioma of WHO-grade 2, who received comprehensive treatment after surgery, has survived for 29 months without recurrence (Table 2).

### Illustrative cases

#### Case 1

**Fig. 1:**
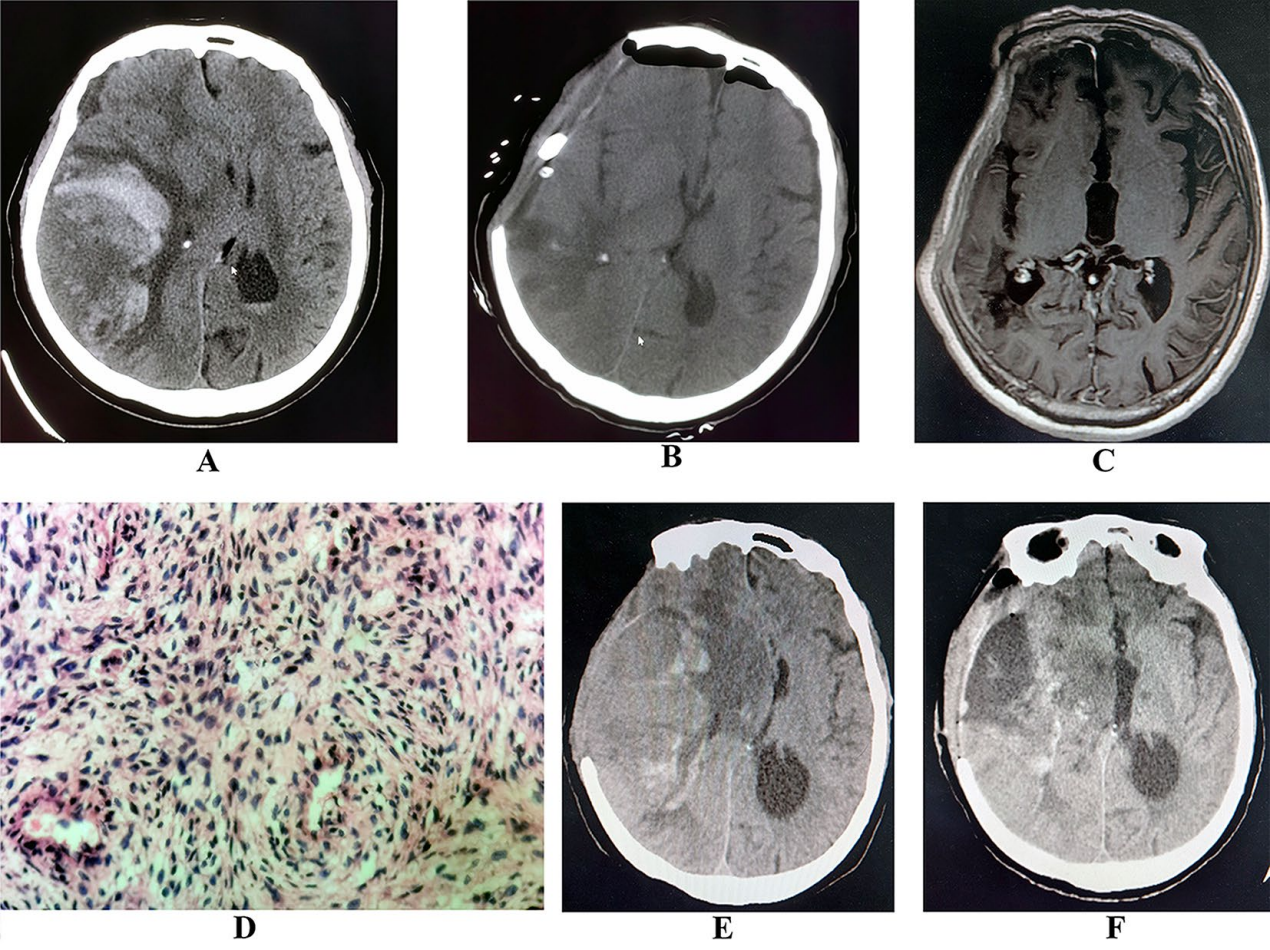
(**A**) The CT of the head after admission, which suggests a right parietal space-occupying lesion with hemorrhage and about a 2 cm shift of the center to the left. (**B**) After admission, an emergency tumor resection + hematoma evacuation + decompressive operation with an osteotomic flap were performed, after which the head CT was reviewed. The midline gyrus is visible, and no tumor or hematoma residue is observed. (**C**) Postoperative re-examination cranial MR showing right parietal glioma postoperative, no residue. (**D**) Postoperative pathology findings: gliosarcoma. (**E**) Two months post-surgery, the repeat head CT shows visible tumor recurrence and hemorrhage. (**F**) CT of the head showing postoperative change in resection of recurrent glioma, basic midline gyration

A 72-year-old female patient was admitted to the emergency department due to “headache, nausea, and vomiting with unconsciousness for 6 hours”, a skull CT (Fig. 1A) showed right temporo-parietal apoplectic intratumoral hemorrhage of glioma. The physical examination findings were as follows: GCS score of 7, bilateral pupils of equal size, about 3.0 mm in diameter, slow reflexes to light stimulation. In the emergency department, craniotomy tumor resection and decompressive removal of the bone flap were performed, and after the operation, the patient was conscious. A review of the cranial MRI showed total resection of the tumor (Fig. 1B). The pathology results showed gliosarcoma of WHO-grade 4 (Fig. 1C).

 The patient was discharged without radiotherapy or chemotherapy. Two months postoperatively, the patient was readmitted to the hospital due to “headache with unconsciousness for 1 day”. The physical examination findings were as follows: GCS score of 4; left pupil diameter 3.0 mm; right pupil diameter 4.0 mm; and no response to light stimulation. A CT scan of the skull (Fig. 1D) showed a recurrent right temporo-parietal tumor with apoplectic intratumoral hemorrhage. Because of the high malignancy of the tumor and its rapid recurrence, family members hesitated but later opted for surgery 48 h after the onset. We performed a tumor resection and hematoma evacuation, and intraoperatively, we observed that the tumor blood supply was rich. Intraoperative blood loss was 1600 ml. The tumor was completely resected.

Postoperative review head CT (Fig. 1E) showed that the right temporoparietal glioma was totally removed and the midline shift was reduced when compared with the condition prior to surgery. The pathology result revealed gliosarcoma. After the operation, the patient continued to be comatose, and the condition was complicated by a pulmonary infection. A tracheotomy was done, and the patient was put on ventilator-assisted breathing and other symptomatic treatment. The patient remained in the same condition without improvement. Twenty days after the operation, the family members chose not to continue further treatment, and the patient died.

#### Case 2

**Fig. 2:**
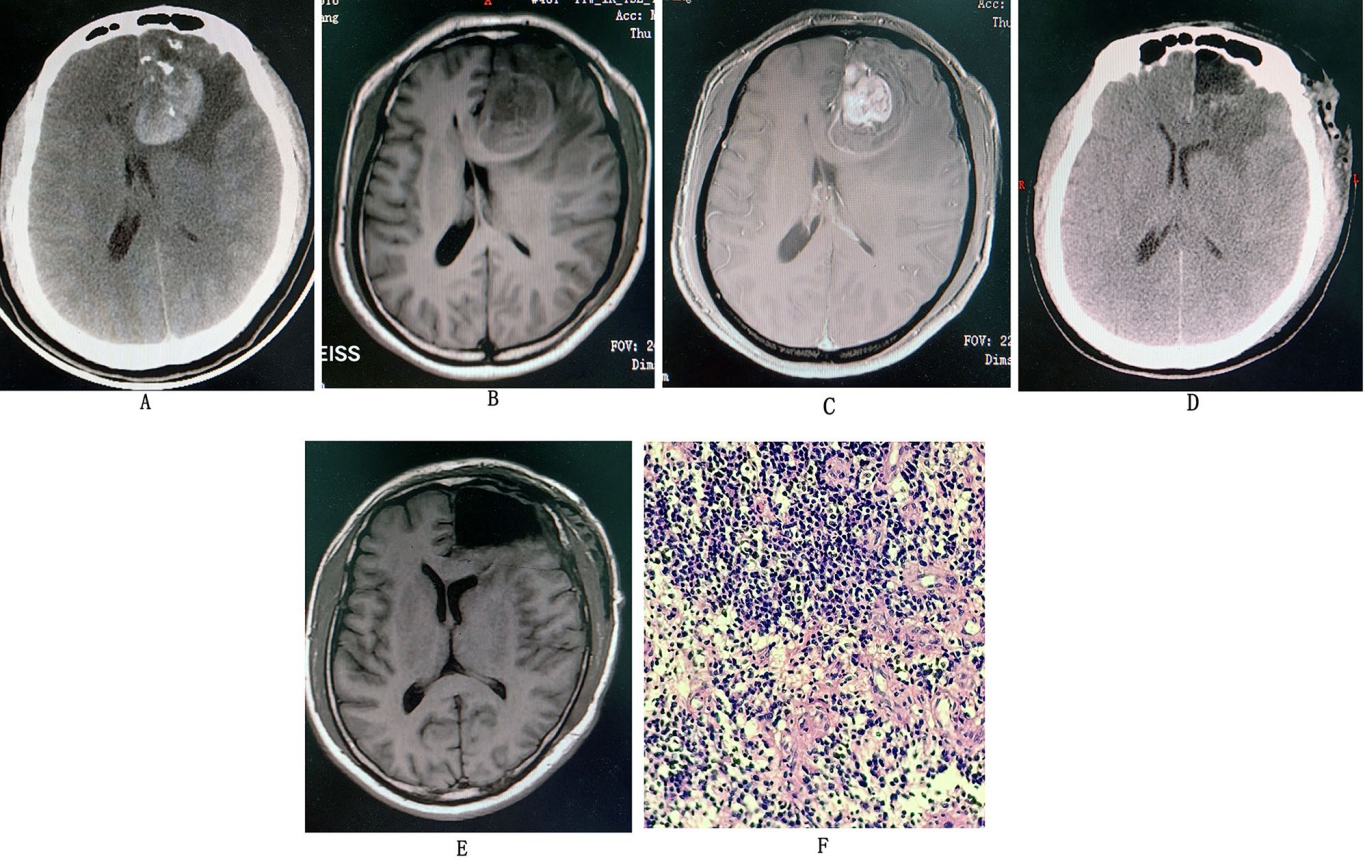
(**A**) Head CT after onset of symptoms in the patient shows a visible left frontal lobe space-occupying lesion with apoplectic intratumoral hemorrhage; to consider glioma complicated hemorrhage. (**B**) Preoperative cranial MRI T1 phase. (**C**) Postoperative MRI enhancement. (**D**) Postoperative CT of the head showing total resection of the tumor with midline gyration. (**E**) Postoperative MRI of the head showing the tumor is totally resected without any residue. (**F**) Postoperative pathology results showing anaplastic oligodendroglioma of WHO-grade 3

A 54-year-old male was admitted to the hospital, reporting a one-week history of headache that had worsened over the past day. Skull CT imaging (Fig. 2A) revealed a left frontal mass characterized by a complex density shadow. The imaging suggested the need to consider possibilities such as glioma and a neutral cerebral hemorrhage associated with stroke. Serial cranial MRI plain scan (Fig. 2B) + enhancement (Fig. 2C) revealed the following findings: an abnormal signal shadow in the left lobe with significant local enhancement, indicative of a glioma with apoplectic intratumoral hemorrhage. The GCS score was 15 points. Subsequently, the patient underwent a craniotomy for tumor resection and hematoma evacuation. Postoperatively, the patient was lucid, and a review of the skull CT (Fig. 2D) and MRI (Fig. 2E) showed a total resection of the tumor. The pathology results (Fig. 2D) indicated an oligodendroglioma of WHO-grade 3. The patient received postoperative chemoradiotherapy. Currently, the patient is in good health overall with a KPS score of 90, and at the 9-month follow-up, there were no signs of recurrence.

#### Case 3

**Fig. 3:**
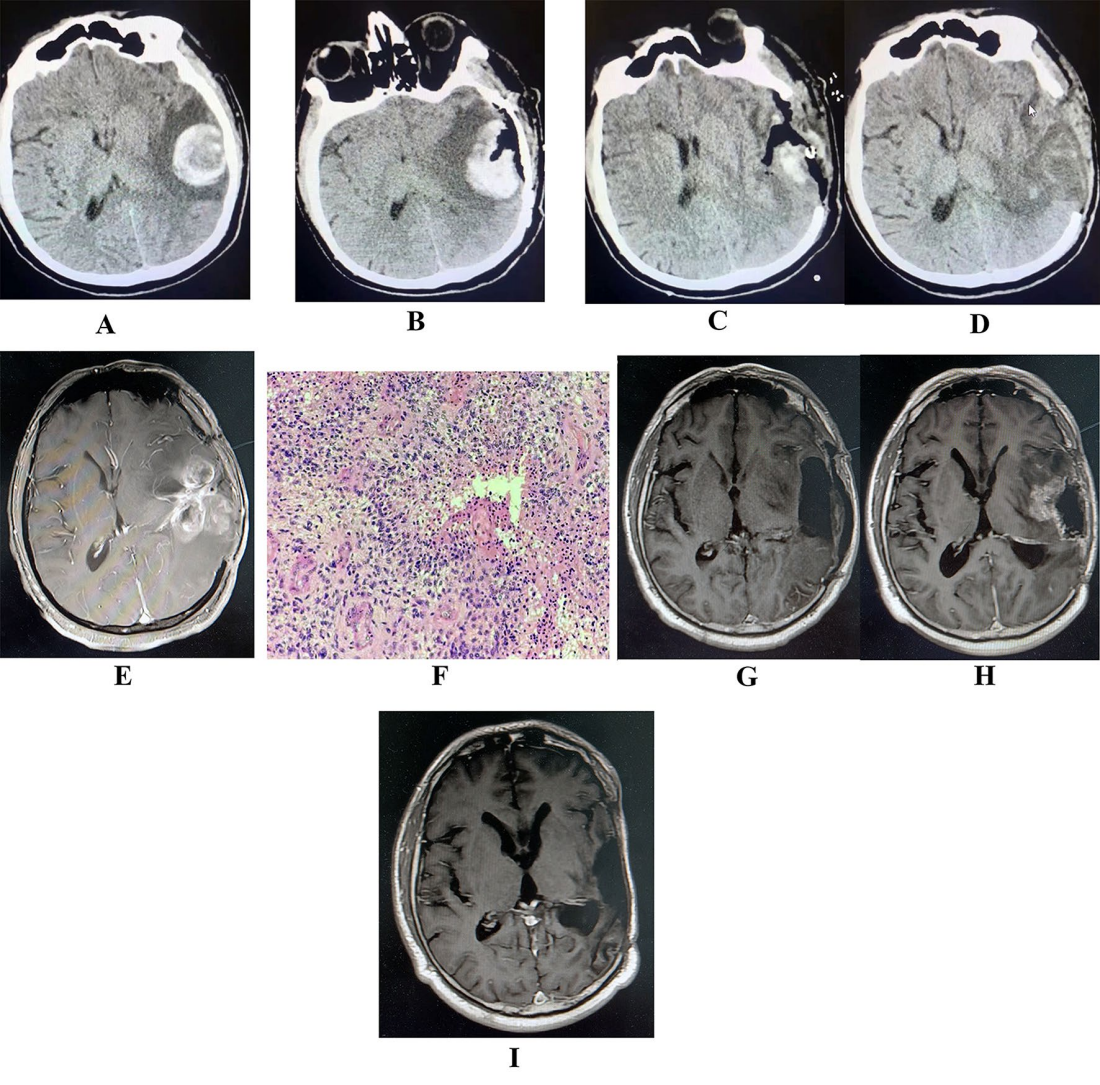
**A**. Head CT performed two hours after the onset of symptoms reveals a left temporoparietal hemorrhage with perihematomal edema. **B**: Twelve hours after the craniotomy hematoma evacuation, the patient remained unconscious, and a follow-up head CT shows a hemorrhage in the operative area, while the brain tissue in the edema area remains unchanged. **C**: Subsequent head CT after a secondary craniotomy shows a clean evacuation of the hematoma. **D**: Twenty days post-surgery, the head CT indicates the emergence of cerebral edema in the left temporoparietal lobe. **E**: A cranial enhanced MRI reveals a space-occupying lesion in the left temporoparietal lobe, indicating a potential high-grade glioma. **F**: The pathological analysis indicates a glioblastoma of WHO-grade 4. **G**: Following the surgical removal of the tumor, a review of the head MRI confirms a complete resection without any residue. **H**: Four months after the glioblastoma resection, a follow-up cranial MRI shows in-situ tumor recurrence. **I**: A cranial MRI, taken after four months of concomitant temozolomide and bevacizumab treatment, shows the disappearance of the primary recurrent glioma

A 50-year-old male visited a nearby hospital due to the sudden onset of a headache accompanied by nausea and vomiting that lasted for four hours. The researcher assessed the GCS score at 11 points. A CT scan of the head (Fig. 3A) revealed a left temporoparietal hemorrhage with perihematomal edema. An emergency craniotomy hematoma evacuation was performed under anesthesia. Twelve hours post-surgery, the patient remained unconscious, and a review of the head CT (Fig. 3B) revealed bleeding in the surgical area. Subsequently, an additional craniotomy was performed for hematoma evacuation and decompressive removal of the bone flap. A postoperative review of the head CT on day 1 (Fig. 3C) indicated a successful evacuation of the hematoma and absence of the bone flap. Following the procedure, the patient regained consciousness and reported relief from the headache.

 However, after 20 days of conservative treatment, the patient once again presented with headache symptoms. A review of the head CT (Fig. 3D) showed unresolved edema of the brain tissue in the surgical area, with signs of possible new organisms. Following this, the patient was transferred to our hospital and underwent a cranial MRI scan (Fig. 3E), which revealed a large abnormally enhancing focus in the left temporoparietal cluster. We considered glioblastoma as a possible diagnosis. Following thorough preoperative preparation, the patient underwent a resection of the left temporoparietal glioma. A postoperative review of the cranial MR (Fig. 3F) confirmed a complete resection of the tumor. Postoperative pathology (Fig. 3G) confirmed the diagnosis of glioblastoma, WHO grade 4.

The patient received concurrent chemoradiotherapy as per the treatment plan. Four months post-surgery, a follow-up cranial MR scan (Fig. 3H) indicated a potential recurrence of the tumor. To address this, chemotherapy with temozolomide combined with bevacizumab was initiated. Four months later, a review of the cranial MR (Fig. 3I) showed the disappearance of the left temporoparietal tumor. Nine months after surgery, the patient remained in a stable general condition with no signs of recurrence. Chemotherapy with a combination of temozolomide and bevacizumab was continued.

## Discussion

### Clinical manifestations

Glioma complicating neoplastic hemorrhage is a relatively uncommon occurrence, with an incidence of approximately 3.7–7.2% among all patients with glioma [4]. This phenomenon is primarily associated with gliomas characterized by rapid growth, higher malignancy, and abundant blood supply, especially high-grade gliomas and oligodendrogliomas [6–8]. Within this patient cohort, pathology findings identified the majority of the tumors as WHO-grade 4 glioblastoma, consistent with reports in the existing literature. Previous studies have indicated that glioblastomas are mostly located in the cerebral hemispheres, with rare occurrences in the spinal cord and thalamus and even rarer instances in the cerebellum [9]. The data from this group of patients are similar to this trend, with all patients with glioblastoma harboring tumors located in the cerebral hemispheres or thalamus.

Currently, it is widely believed that the main causes of AIH in brain tumors can be attributed to the following factors: The hemorrhage risk in higher-grade brain tumors is related to malignancy, characterized by rapid tumor cell proliferation, vascular invasion, neovascularization, and necrosis [10]. Conversely, in low-grade tumors, the etiology of hemorrhage may be related to structural abnormalities and specific vascular architecture of tumor vessels [11].

Due to the tumor’s high malignancy and fast growth, most patients experience a combination of symptoms related to high cranial pressure. They often have a history of intermittent cephalalgia. However, if a patient presents with comorbid glioma and apoplectic intratumoral hemorrhage, along with a significant increase in cranial pressure, the onset is typically acute, and the condition is severe. In such cases, emergency admission to the hospital is typically warranted.

### Differential diagnosis

Glioma with apoplectic intratumoral hemorrhage is sometimes easily misdiagnosed as simple hypertensive cerebral hemorrhage or cerebral vascular malformation hemorrhage, thus delaying appropriate treatment. Cranial CT scans are mostly used to help differentiate the apoplectic intratumoral hemorrhage of gliomas from simple intracerebral hemorrhage [12]. The location of gliomas combined with hemorrhage is identified using the location of the tumor, and hypertensive cerebral hemorrhages are typically located in the basal ganglia.

The characteristics of glioma with hemorrhage diagnosed using a CT scan were: (1) The location of the hematoma could be localized with the site of the tumor and was mostly situated in the cerebral lobes. (2) Hemorrhage foci showed uneven density or irregular shape. (3) Peritumoral edema did not coincide with the duration of edema induced by intracerebral hemorrhage, and cerebral edema could occur after hemorrhage from a tumor stroke, which is referred to as tumoral edema [12]; Hypertensive ICH generally presented with edema three hours after hemorrhage, which peaked at 48 h and subsequently resolved gradually. (4) The tumor body, necrosis foci, and enhancement of the posterior tumor wall or the occurrence of enhancement of the tumor body could be observed on the side of the hemorrhage. (5) In a glioma-related stroke, even small hemorrhages can have a significant space-occupying effect, often causing a midline shift and compression of the cerebral ventricles. Even after blood absorption, there could still be a significant space-occupying effect, considering the cytotoxic brain edema related to the tumor. In cases of hypertensive intracerebral hemorrhage, the edema tended to reduce after absorption of the treated hematoma.

While MRI is popular due to its good tissue resolution and is considered superior to CT for localizing and visualizing gliomas with hemorrhage, it is particularly valuable for the differential diagnosis of this disease [13]. Patients within this category should undergo cranial MRI with gadolinium enhancement, provided their clinical condition allows for undergoing the procedure. This imaging modality can help reveal both tumor signs and signs of hematoma, as well as identify heterogeneous enhancement in the non-hemorrhagic portion of the tumor. Such enhancements can distinguish the tumor from a simple intracranial hematoma and guide the optimal extent of surgical resection.

The focus of our study cohort was on patients in primary hospitals. Five patients were initially diagnosed with only intracerebral hemorrhage in primary hospitals, and they were treated according to this diagnosis. However, their high cranial pressure could not be relieved. Upon undergoing a cranial MRI, these patients were found to have glioma with hemorrhage. This highlights the importance of an accurate preoperative diagnosis.

Furthermore, one patient in our cohort had a brain hemorrhage that extended into the ventricular system. Head CT scans indicated that peritumoral edema was not apparent, and angiography ruled out arteriovenous malformation as the cause of the hemorrhage. Finally, a cranial MRI was performed to establish a definitive diagnosis. The small size of this particular tumor type had not yet caused obvious space-occupying effects. In other words, the hemorrhage occurred in ruptured vessels, and this could have easily been misdiagnosed. This scenario also reflects the higher malignancy of this class of tumors, as necrosis had already occurred in the core part of the tumor or the vessel wall had been invaded, resulting in hemorrhage.

As a result, it is imperative that patients with intracerebral hemorrhage in uncommon locations undergo cranial MRI with plain and contrast-enhanced examinations. To avoid misdiagnosis, cerebrovascular disease and hypertensive intracerebral hemorrhage should be ruled out first. If the challenge of accurate identification persists, then choline PET/CT can be considered, as it has a high value in differential diagnosis [14].

### Timing of surgery and precautions

If the GCS score is < 9, the patient is considered critically ill and requires immediate surgical management. If a patient has a GCS score of ≥ 9, it warrants a sub-emergency operation, and the patient still requires expedited preoperative preparation. This is because some patients can develop successive hemorrhagic events during the waiting period, which can lead to secondary brain damage and increased morbidity. However, once re-bleeding aggravates the condition of patients in coma, emergency treatment is required, and the treatment effect is greatly reduced. Hence, early intervention is mandated to be performed as far as possible, and our observation with the cohort in this study is consistent with literature reports [15, 16]. However, there is not much information available regarding the short-term probability of rebleeding among patients in this category [15]. In our cohort, one patient experienced secondary hemorrhages during the waiting period, which affected the brain function, caused a coma, and adversely impacted the prognosis.

Obstructive hydrocephalus occurs due to massive hemorrhage or hemorrhage breaking into the ventricles or compression of the cerebrospinal fluid circulation pathway by the tumor and hemorrhage in some patients with glioma stroke, and the condition deteriorates quickly, necessitating urgent surgical management. In our patients, fluid planes were visualized in the cystic glioma cavity with hyperdense blood in the lower half and hypodense fluid in the upper half, which are typical imaging findings of cystic glioma with hemorrhage. In this category of cystic gliomas with hemorrhage, patients require urgent surgical intervention to relieve the herniation. Additionally, bedside ventricular puncture with external drainage is feasible, taking care to ensure that the solid part of the tumor is avoided during the puncture and the cystic fluid is gradually released to avoid inducing rebleeding. Before the procedure, the patient’s family should be informed about the associated risks, such as puncture failure, successive hemorrhages, and so on.

However, in cases of solid gliomas complicated by hemorrhage and critical illness, hematoma puncture and external drainage are generally not recommended; an emergency craniotomy with hematoma evacuation and tumor resection are necessary. If intraoperative tumor tissue is not discernible and intraoperative frozen definitive pathology is feasible, it is used to decide the extent of surgical resection. If the tumor has a clear boundary with surrounding normal brain tissue that can be clearly discerned microscopically, the gross total resection is then routinely sent for pathology examination.

Intraoperative ultrasound probing can also be used to locate tumor tissue and better guide the extent of resection [17]. Intraoperative evacuation of hematoma, complete resection of tumor tissue, protection of vital neurological function, and adequate intracranial decompression are extremely important for the prognosis of patients [18, 19]. Because most cases in this category were high-grade gliomas in our cohort, the extent of resection was slightly extended when necessary. The decision on whether to refund or discard a bone flap is based on the patient’s preoperative consciousness and intraoperative cranial pressure. Decompression of the bone flap, if necessary, proves advantageous in the postoperative management of critically ill patients and can be life-saving for patients who are critically ill [20].

If a patient presents with tumor recurrence, reoperation can still prolong patient survival as long as the patient’s overall condition is satisfactory, especially if the patient is less than 60 years old and the KPS score is greater than 70 [21, 22]. In our cohort, there were three patients with glioma recurrences who underwent second surgeries. These included a patient with a preoperative GCS score of < 9 who died perioperatively; two other patients, who both had a preoperative GCS score of ≥ 9 points, were discharged uneventfully, and their survival was prolonged.

### Subsequent treatment after surgery

Postoperative interventions were based on the pathology results and included routine radiotherapy and chemotherapy. Chemotherapy was administered with a single-agent temozolomide regimen or a regimen of temozolomide plus bevacizumab. The combination of temozolomide and bevacizumab has been reported to have better efficacy and safety in the treatment of high-grade gliomas [23–25]. One patient in our cohort experienced tumor recurrence after single-agent chemotherapy with temozolomide. We followed this with two months of chemotherapy combined with bevacizumab, and the recurrent tumor disappeared, indicating that bevacizumab played an important role. In instances involving larger recurrent tumors, secondary surgical intervention may be extended as a viable option, provided that the patient does not present surgical contraindications. Furthermore, the prospect of prolonged survival aligns with this therapeutic approach.

### Prognostic factors

Patients with this category of tumors are doubly burdened due to the combination of high glioma malignancy and tumor hemorrhage, which poses significant challenges in treatment. Unfortunately, the majority of these patients have a poor prognosis and shorter survival.

We consider three factors playing a crucial role in determining the prognosis. Firstly, the patient’s preoperative state of consciousness is of utmost importance. The space-occupying effect caused by the size of the tumor and the volume of hemorrhage, as well as the resulting damage to brain tissue, determines the patient’s level of consciousness. Patients with a preoperative GCS score of ≥ 9 tend to have a relatively favorable prognosis. Conversely, a preoperative presentation characterized by severe consciousness disturbance and a GCS score < 9 is generally indicative of an unfavorable prognosis.

Secondly, the location of the tumor and the extent of resection are important factors influencing the prognosis. The prognosis is relatively good if the tumor is situated in a nonfunctional area and is completely excised while the surrounding vital structures, such as the thalamus, brain tissue in the functional area, normal peritumoral arteries, and veins, are preserved intact. However, the removal of gliomas located in deeper regions of the brain, such as the thalamus and corpus callosum, poses challenges during surgery, making total resection difficult. These cases are associated with a severe postoperative response and typically have a poor prognosis.

Finally, the pathologic grade of the tumor and subsequent treatment options also influence the prognosis. Subsequent treatments for glioma, such as postoperative chemoradiotherapy, are planned based on the pathological results and the patient’s physical condition. In the scenario of tumor recurrence, there exists the option to modify the chemotherapy regimen or alternatively contemplate a reoperation. Nevertheless, owing to the elevated malignancy rate of the tumor, its rapid recurrence, and the consequential detrimental effect on the confidence of both patients and their families in the treatment process, certain individuals may elect to pursue a relatively conservative form of elimination therapy subsequent to tumor recurrence. Consequently, the prognosis for the disease in such cases tends to be generally unfavorable.

## Conclusions

Although rare, apoplectic intratumoral hemorrhage of glioma (AIHG) has a rapid onset and can result in severe neurological dysfunction. The dual complications of glioma and intracerebral hemorrhage pose a challenge in clinical management. Therefore, in suspected cases identified through CT scans, a head MRI scan must be done at the earliest possible time while vital signs are stabilized. Early surgical intervention following a definitive diagnosis is crucial. Intraoperative procedures should aim for complete tumor resection, with the removal of intracranial hematoma as well. Additionally, careful postoperative management is necessary to ensure successful recovery during the perioperative period.

For patients with a Glasgow Coma Scale (GCS) score of ≥ 9, combining tumor resection with hematoma evacuation can yield treatment outcomes comparable to elective surgery for gliomas of the same pathological grade. However, patients with a GCS score < 9 will require immediate surgery to alleviate herniation and remove the glioma. Unfortunately, these cases often have a poor prognosis. The overall prognosis of the disease is influenced by factors such as tumor size, location, site and volume of hemorrhage, preoperative consciousness status, and histopathological findings. Overall, treating this disease is challenging, with a bleak long-term prognosis.

## Data Availability

The datasets used and/or analysed during the current study available from the corresponding author on reasonable request.

## Abbreviations

GCS: Glasgow Coma Scale
AIHG: Apoplectic intratumoral hemorrhage of glioma
MRI: Magnetic Resonance Imaging
